# Contamination of Runoff Water at Gdańsk Airport (Poland) by Polycyclic Aromatic Hydrocarbons (PAHs) and Polychlorinated Biphenyls (PCBs)

**DOI:** 10.3390/s111211901

**Published:** 2011-12-20

**Authors:** Anna Maria Sulej, Żaneta Polkowska, Jacek Namieśnik

**Affiliations:** Chemical Faculty, Gdańsk University of Technology, ul. Gabriela Narutowicza 11/12, 80-233 Gdańsk, Poland; E-Mails: zanpolko@pg.gda.pl (Z.P.); chemanal@pg.gda.pl (J.N.)

**Keywords:** airport runoff water, PCBs, PAHs, pollutants

## Abstract

Airport runoff can contain high concentrations of various pollutants, in particular polycyclic aromatic hydrocarbons (PAHs) and polychlorinated biphenyls (PCBs), the environmental levels of which have to be monitored. Airport runoff water samples, collected at the Gdańsk-Rębiechowo Airport from 2008 to 2009, were analysed for PAHs and PCBs by gas chromatography. The aromatic fractions were separated by liquid-liquid extraction and analysed by GC/MS. Total PAH concentrations were 295–6,758 ng/L in 2008 and 180–1,924 ng/L in 2009, while total PCB levels in 2008 ranged from 0.14 to 0.44 μg/L and in 2009 from 0.06 to 0.23 μg/L. The PAH and PCB compositions in airport runoff waters were examined over a range of spatial and temporal scales to determine distributions, trends and possible sources. This pollution is mainly pyrolytic and related to anthropogenic activity. There were significant differences between the samples collected in the two seasons. An understanding of the magnitude of contamination due to airport runoff water is important for the effective management of airport infrastructure.

## 1. Introduction

Despite the many positives emerging from the rapid expansion of the air transport sector, all activities relating to airport operations lead to environmental contamination [1–4]. Increases in aviation developments have serious consequences on the surrounding air, soil and water bodies like lakes, rivers and ground water [5]. The management of local water and air quality is a significant environmental issue for many airports.

Wet deposition is one of the most important routes through which atmospheric pollutants reach the surface of the Earth. Atmospheric pollutants are scavenged to the largest extent during the initial stages of the precipitation event (first flush), and their concentration decreases exponentially with time [6]. This was observed among others in studies performed in Germany and Switzerland, in which the highest concentration of pollutants were observed in the initial portions of precipitation and runoff waters. Then, the rainfall waters and arising from them runoff waters, together with various toxic compounds, get into the soil, surface waters and groundwaters, which can be the source of drinking water [7–9].

A particularly important problem in airports managements is the contamination caused by airport runoff waters. Much of the land surface in urban areas is impervious, and does not allow rain and snowmelt to soak into the ground, thus contributing to the increase of runoff volume. Airport runoff waters are formed when rain or melting snow washes off the airport apron the chemicals used for deicing and antiicing, refuelling, vehicle cleaning and maintenance, as well as the products of fuel combustion and spilt fuel. Both precipitation and runoff waters get into the surface waters and even ground waters. Two particularly hazardous groups of chemicals that can enter the environment along with airport runoff waters are polycyclic aromatic hydrocarbons (PAHs) and polychlorinated biphenyls (PCBs) [10–13].

In recent years, pollution by PAHs has led to numerous studies of their origin, distribution and fate in the environment. The sources of emissions of these compounds are numerous. PAHs released into the environment are mainly anthropogenic, and it is currently assumed that PAHs are mainly of pyrolytic origin. In addition, PAHs enter the environment through contamination by crude oils, coal, coal tar or various refinery products (petrogenic origin) [14] and natural sources like forest fires and volcanic activity [15,16].

PCBs also have a destructive effect on the environment. Their concentration in the environment, especially in rain and urban storm water, is presumed to have declined due to banned use, but despite this, PCBs are present in practically all environmental samples, sometimes at surprisingly high levels. PCBs can also be present in airport runoff waters [17,18]. Those present in airport storm water runoff are associated with specific activities, e.g., they were used as heat exchanger fluids, chemical stabilizers, and in hydraulic systems in the aluminium, copper and iron smelting industries. PCBs were also used as plasticizers in natural and synthetic rubber products like adhesives, installation materials, flame retardants, lubricants, chemical stabilizers in paints, pigments and oil varnishes at airports [19].

PAHs and PCBs are particularly hazardous to all compartments of the environment because they are highly toxic and carcinogenic [20–22]. In view of this it is essential to draw greater attention to assessments of the effect of runoff on different compartments of the environment and on living organisms [23].

To date, PAH levels have been determined in only a few environmental samples, for example, in air samples from airports in Italy (99–114 ng/m^3^ [24]) and California (av. 18–570 ng/m^3^ [25,26]). PAHs were also determined in soil samples from Delhi International Airport (India) (1.7–7,529 μg/kg). The PAH compound most commonly determined was benzo(a)pyrene, with soil concentrations of 1.0–530 μg/kg [21,27]. No analysis has been done of airport runoff waters for the presence of PCBs. But the detection and quantitative determination of PCBs has been carried out in samples of water and bottom sediments from the river in the vicinity of Washington (USA) airport. The levels of these pollutants ranged from not detected to 22 μg/L in the river water and from 0.05 to 10,000 (mg/kg dry weight) in the bottom sediments [28].

To date relatively little information is available on the results of analyses of airport runoff waters. Nevertheless, this type of material object is attracting increasing attention as a source of information about its potential adverse effect on the environment [28–32]. The present work deals with the determination of PAHs and PCBs found in airport runoff waters from the Lech Wałęsa Airport in Gdańsk, Poland. The PAH and PCB concentrations and distributions are discussed in terms of sampling location, seasonal variation, origin and sources.

## 2. Experimental Section

### 2.1. Sampling

**Figure 1.**
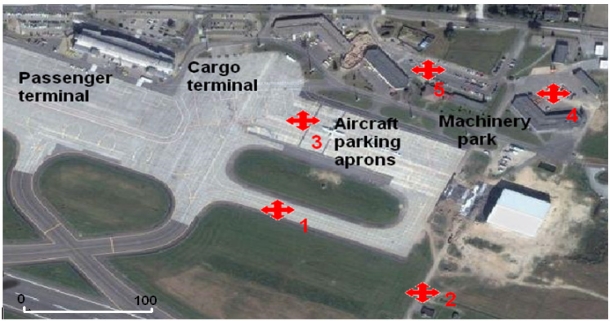
Site description of the Lech Wałesa Airport at Gdańsk Rębiechowo.

The samples of runoff and precipitation water were collected during or shortly after the rainfall (depending on its intensity, at least 2–3 mm) at the Lech Wałęsa Airport in Gdańsk, the third-largest airport in Poland in three seasons—winter, spring and summer—from 2008 to 2009. They were taken in series of 25 in each season from small depressions. The sampling locations are areas of the airport with the greatest concentration of technical service operations: aircraft refuelling, loading and unloading (cargo aircraft), deicing, parking and servicing of ground staff vehicles. These are therefore sites where the largest amounts of pollutants enter drainage ditches with runoff and then into the environment at large. The sampling locations on Airport Gdańsk are labelled in Figure 1 and described in Table 1.

Atmospheric precipitation samples were collected during or immediately after a precipitation event. Rain samples were collected in 250 cm^3^ dark bottles equipped with glass funnels. Airport runoff samples at each location were collected in 1,000 mL bottles of dark glass using plastic tubes mounted at the edge of the asphalt surface to ensure free flow of the sample to the collection vessels. The samples were transported to the laboratory (usually within less than 1 h of collection. Prior to use, the syringes and tubing were rinsed with MilliQ water and then with the water to be sampled. As the runoff samples were usually highly contaminated with solids (sand, leaves, *etc*.), they had to be pre-filtered (0.45 μm, Millex®-HV). Bottles were stored at 4 °C in the dark until extraction [33–37].

### 2.2. Chemicals

The organic compounds investigated were PCBs (PCB 28, PCB 52, PCB 101, PCB 118, PCB 153, PCB 138, PCB 180) and PAHs (naphthalene, acenaphthylene, acenaphthene, fluorene, phenanthrene, anthracene, fluoranthene, pyrene, chrysene, benzo(b)fluoranthene, benzo(k)fluoranthene, benzo(a)pyrene, benzo(a)anthracene, indeno(1,2,3-cd)pyrene, dibenz(a,h)anthracene), benzo(g,h,i)perylene. Predeuterated aromatic compounds (naphthalene-d8, benzo(a)anthracene-d12) and mixtures of 16 PAHs (2,000 μg/mL in dichloromethane) from Supelco (Bellefonte, PA, USA) and Restek Corporation (Bellefonte, PA, USA), respectively, were used as internal standards. A working stock solution was prepared from seven selected PCB standards, IUPAC Nos. 28, 52, 101, 118, 153, 138 and 180 (10 μg/mL in isooctane) from Restek Corporation. Certified standards of 13C-labelled PCB 28 and PCB 180 (40 μg/mL in nonane) were obtained from Cambridge Isotope Laboratories (Warsaw, Poland). All solvents used for sample processing and analyses were GC-pure quality and were purchased from Merck (Darmstadt, Germany).

### 2.3. Analytical Procedure

**Figure 2.**
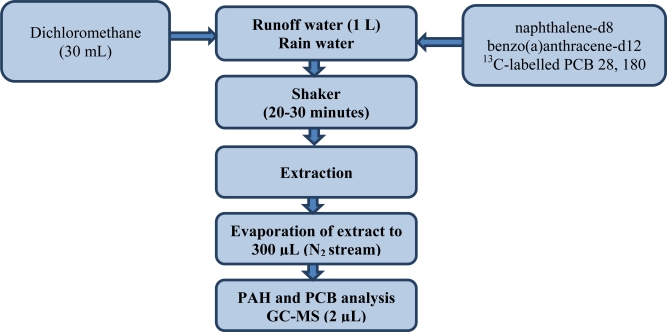
Flow chart of the procedure for preparing runoff water and rain samples for determining their PAH and PCB contents.

Approximately 1 L of samples was liquid-liquid extracted with 30 mL dichloromethane, two isotopically labelled internal standards of PCB 28 (270, 268 *m/z*) and PCB 180 (408, 406 *m/z*) and two deuterated internal standards: naphthalene-d8 (136 *m/z*) and benzo(a)anthracene-d12 (240 *m/z*) and shaken for 20–30 min. Internal standards were used for quality control. After liquid-liquid extraction (LLE) the extract was evaporated to a volume of 300 μL under a gentle stream of nitrogen. The runoff water samples so prepared were analysed by GC-MS. This organic analysis included 16 polycyclic aromatic hydrocarbons (PAH): naphthalene, acenaphthylene, acenaphthene, fluorene, phenanthrene, anthracene, fluoranthene, pyrene, benzo(a)anthracene, chrysene, benzo(b)fluoranthene, benzo-(k)fluoranthene, benzo(a)pyrene dibenz(a,h)anthracene, benzo(ghi)perylene and indeno(1,2,3-cd)-pyrene and seven polychlorinated biphenyls (PCB) IUPAC Nos. 28, 52, 101, 118, 138, 152, and 180. This procedure for determining PAHs and PCBs in water samples was carried out on the basis of the procedure described by ISO 17993 standard. It was subsequently modified in that a GC-MS system was used for the final determinations [38–42]. Figure 2 shows a flow chart for the determination of PAHs and PCBs in samples of sea water. The experiments were performed using an Agilent Technologies 7890A gas chromatograph with an Agilent Technologies 5975C mass spectrometric detector and split/splitless injector. Table 2 gives information on the GC-MS operating conditions.

Mass detection was performed in the selected ion monitoring (SIM) mode. The following mass to ion ratios were monitored: (*m/z*) PAH: 128, 127, 152, 151, 153, 154, 166, 165, 178, 176, 203, 202, 228, 226, 252, 250, 277, 276, 278, and 279, and PCB: 256, 258, 290, 292, 234, 326, 358, 360, 392, and 394. Before sample analysis, the relevant standards were analysed to check column performance, peak height and resolution, and the limits of detection and quantification. A solvent blank, a standard mixture and a procedural blank were run in each sequence of samples to check for contamination, peak identification and quantification. Compounds were identified mainly by their retention times. Measuring range, detection and quantification limits are presented in Table 3.

## 3. Results and Discussion

### 3.1. Total Concentrations and Relative Distributions of PAH

**Figure 3.**
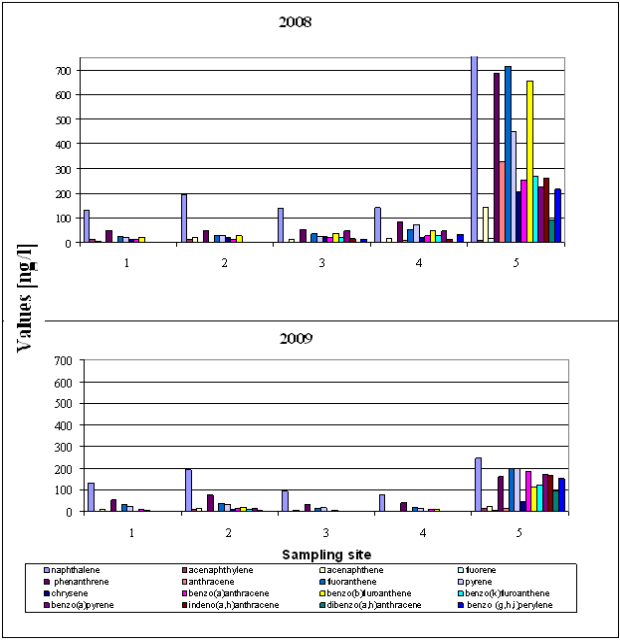
Concentration of PAHs determined in runoff water taken from five sampling locations at Gdańsk Airport (2008–2009).

Figure 3 shows the measured concentration of PAH determined in samples of runoff water collected from five locations at Gdansk Airport in 2008–2009; the PAH distribution was the same at most of the sampling sites in both 2008 and 2009.

This uniformity and the permanence of the total concentrations suggest a similar source of the PAHs in most of the samples. The sum of 16 PAHs (Σ PAH) ranged from 295 ng/L to 6,758 ng/L in 2008, and from 180 ng/L to 1,924 ng/L in 2009. The mean value of ΣPAH for the five sampling locations was around 1,703 ng/L in 2008 and 601 ng/L in 2009. Generally, PAH concentrations in the 2008 samples were higher than those in the samples from 2009. The next important feature is the predominance of naphthalene at all locations in 2008 and 2009 (Figure 3). Of all the PAHs in crude oil, naphthalene was present in the largest amounts and can therefore be used as an indicator substance for assessing whether an oil spill has occurred or not. The data in Figure 3 indicate that fuels spills did occur at all the sampling sites in both years of the study. PAH levels were the highest at Site 5. This is because rain water flushes pollutants off the concrete apron surface which then get into the environment around the edges of the airport with runoff water—there is no waste water treatment plant at this airport. Site 5 is additionally burdened with runoff from the road leading up to the airport entrance gate.

**Figure 4.**
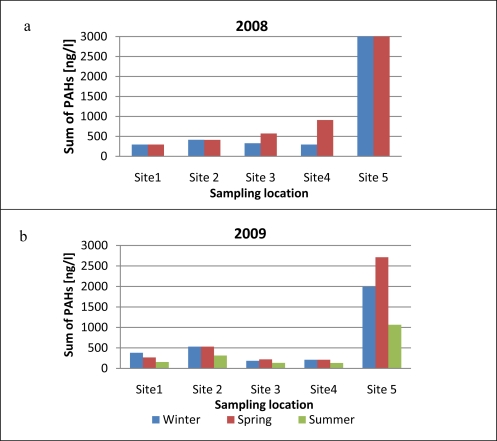
Seasonal changes in total PAH concentrations in runoff water samples collected in 2008–2009.

The seasonal changes in the sum of PAH concentrations in runoff samples collected in 2008–2009 from different locations on Gdansk Airport are shown in Figure 4. Comparison of the sum of PAHs in 2008 and 2009 shows that PAH levels were significantly higher in 2008 than in 2009. Moreover, the smallest amounts of PAHs were emitted in summer than in other seasons. Total PAH concentrations in winter 2009 were 1.8 times higher than in summer, and in spring 2009 they were 2.2 times higher than in summer. In spring 2008 [Figure 4(a)] PAH concentrations at all sampling sites were much higher than the winter values. Such high levels of PAHs in early spring samples could be due to the thaw typical of this time of the year, when contaminants accumulated in the frozen airport aprons are flushed out by melting snow and heavy rain. Throughout the research period the highest PAH levels were recorded at site 5 (the airport entrance gate). These contaminants may be carried onto the airport premises by technical service vehicles and by cars bringing passengers to the airport.

### 3.2. Diagnostic Ratio Analysis

Table 4 sets out literature information on the values of parameters that can be used to determine the origin of PAH compounds. Identifying the origin of PAH compounds in airport runoff waters is extremely important: without this information it is not possible to evaluate the extent of their interaction with the aquatic environment or to take appropriate action with regard to their biogdegradation (PAHs derived from combustion processes are more strongly bound to sediment particles and their biodegradation is therefore more difficult). Compounds of diagnostic significance are always those with a similar molecular mass. Such compounds have a similar environmental fate (adsorption, desorption, *etc*.), and likewise, their recoveries at the extraction stage prior to quantitative determination are also similar.

**Figure 5.**
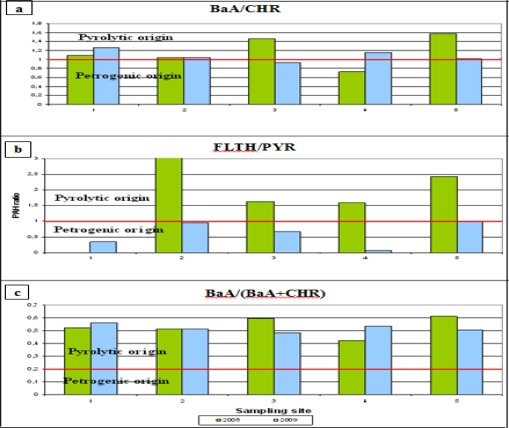
PAH isomer ratios for source indicators: (**a**) BaA/CHR; (**b**) FLTH/PYR; (**c**) BaA/(BaA + CHR). PAH isomer ratios were calculated for five sampling sites at Gdańsk Airport in 2008–2009.

The nature of the emission source of PAHs can be defined using calculated values of indices expressing the concentration ratio of particular PAHs. During combustion processes the alkyl derivatives of PAHs are the less stable thermodynamic form; hence the concentration ratio of alkylphenanthrenes to phenanthrenes is used to show whether combustion processes, and also spills and the processing of crude oil, are sources of PAHs. The usual values of the indices (FLTH/PYR > 1, BaA/CHR > 1, BaA/(BaA+CHR) > 0.2—see Figure 5) indicate that combustion is responsible for the PAHs present in samples of airport runoff. In the case of all the runoff samples collected at all five sites on the airport in 2008, the value of FLTH/PYR [Figure 5(b)] indicated quite conclusively that the PAHs were of pyrolytic origin [14], whereas in 2009 the value of FLTH/PYR for all samples was <1, which points to the petrogenic origin of the PAHs [14,43].

According to previous studies, the value of the parameter BaA/CHR > 1 [Figure 5(a)] indicates pyrolytic sources, including incomplete combustion of fuels, while BaA/CHR < 1 indicates petrogenic sources, including spilt petroleum products or oil [50]. In most of the samples for which the PAHs were shown to be of pyrolytic origin, only the samples from the machinery park (2008) and the passenger terminal (2009) were of petrogenic origin (spilt fuel during refuelling, aircraft and ground service vehicle repairs; see Figure 5).

According to the data presented in Figure 5(c), the PAH ratio BaA/(BaA + CHR) of > 0.2 indicates pyrolytic sources, whereas BaA/(BaA + CHR) < 0.2 indicates petrogenic sources. The BaA/(BaA + CHR) ratios in 2008–2009 were generally higher than 0.2 at all the sampling sites. This result suggests the dominance of pyrolytic sources of PAHs in airport runoff waters in those years.

**Figure 6.**
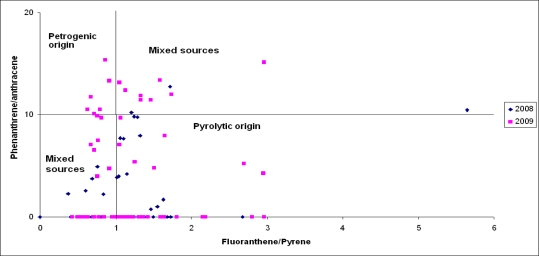
Cross-plot of phenanthrene/anthracene ratios against fluoranthene/pyrene ratios for airport runoff water sampled at different locations.

A cross-plot of the PHE/ANTH ratio *vs.* the FLUO/PYR ratio (Figure 6) indicates a high variability, most likely due to the intensive mixing of various PAH sources during transport and the deposition of PAHs. We selected the FLUO/PYR ratio for source discrimination because it covers gaseous as well as particle-transported PAHs. These compounds are characterized by similar boiling points, vapour pressures, photo-oxidation properties as well as octanol/air and plant/air-partitioning coefficients [59,60]. Hence, the cross-plot of the PHE/ANTH ratio against the FLUO/PYR ratio calculated for the 2008 samples generally indicates pyrolytic sources (62.5%) and mixed sources (37.5%), while the studies in 2009 suggest that PAHs in airport runoff waters were mainly of mixed (56.7%) and pyrolytic origin (23.3%).

The percentage relationship between pyrolytic, petrogenic and other sources of PAH emissions (shown in Table 5), needed for assessing the origin of PAHs found in the runoff samples from Gdansk Airport, suggest that fuel combustion is the main source of PAH emissions.

### 3.3. Correlations of PAH’s Concentration in Runoff vs. Precipitation

Average concentrations of particular analytes of PAHs determined in rainwater and runoff water for samples collected in 2008 from Gdansk Airport area are shown in Table 6. The values of time averaged concentrations of the PAHs taken for calculations were determined for samples collected at a given site at the same time for both the rainfall and the runoff.

The conducted analysis showed that 11 compounds from the PAHs group (naphthalene, acenaphthene, phenanthrene, anthracene, fluoranthene, pyrene, benzo(a)anthracene, chrysene, benzo(b)fluoranthene, benzo(a)pyrene, indeno(1,2,3-cd) pyrene) were determined in the runoff water samples collected in winter in 2008. However the analysis of rainfall water samples collected at the airport at the same time indicated the presence of only five compounds from the group (naphthalene, acenaphthene, phenanthrene, fluoranthene, benzo(g,h,i) perylene).

Generally, the quantitative analysis showed that the concentration levels of PAH compounds in runoff water samples collected in winter were significantly greater (about 5–8 times for naphthalene, acenaphthene, phenanthrene) and up to ca. 52 times higher for fluoranthene, compared with their concentrations determined in rainfall water samples.

The concentration level of pollutants from the group of PAH compounds also depends on the location of the sampling sites, from which runoff water samples were collected. Both in winter and spring, the highest concentration levels of the sum of PAHs were determined for the measurement point No. 5 (entry gate to the airport premises).

Determination of the analytes from the group of PCBs and PAHs during the spring season in the rainfall and runoff water samples showed no significant differences in the concentration levels of the compounds under investigation. However, the concentration of the sum of PAHs determined in runoff water samples was approximately 1.5 times higher in the winter season, compared with the spring one. Generally it can be noticed that the levels of pollutants from the group of PAHs in the samples collected in winter are much higher in comparison with the entire research period.

### 3.4. Total Concentrations and Relative Distributions of PCBs

The average PCB concentrations found in airport runoff water are given in Table 7. The overall concentration of 7 PCBs (ΣPCB) ranged from 0.14 to 0.44 μg/L in 2008 and from 0.06 μg/L to 0.23 μg/L in 2009. The highest PCB levels were determined in samples taken in 2008 from near the passenger terminal.

**Figure 7.**
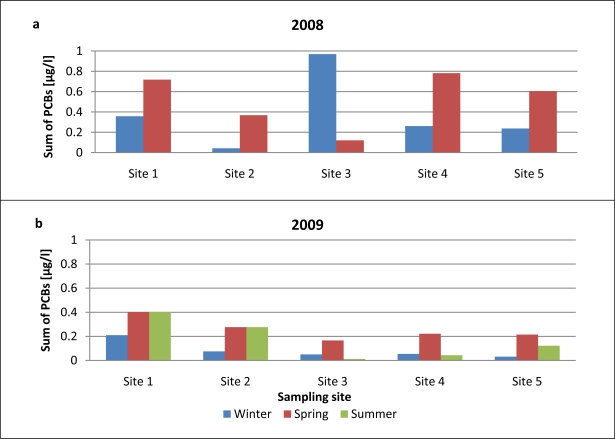
Seasonal changes in overall PCB levels in samples of airport runoff water in 2008–2009.

Figure 7 presents information on the seasonal variations of PCB concentrations in airport runoff water samples collected from the various sites on the airport premises. It is clear from these data that PCB levels were much higher in 2008 than in 2009. In contrast, the average sum of PCBs in runoff waters collected at all 5 sites in spring was much higher than the corresponding winter values. Site 3, the airport apron close to the passenger terminal, was an exception, however: the average PCB concentration in winter samples there was much higher than that in spring runoff samples. Indeed, PCB levels at this site were the highest of all throughout the measurement campaign in 2008–2009. The accumulation of PCBs at this site, immediately adjacent to the passenger terminal, may be due to the more intense service vehicle traffic there; these vehicles very probably carry this kind of contaminant. PCB levels in runoff water samples in 2009 [Figure 7(b)] were comparable to those obtained in 2008. Total PCB concentrations in spring 2009 at all the airport sites were higher than in the winter and summer of that year (spring 2009 was very cold and snowy); consequently, more fuel was consumed, and other operations supporting flight safety (deicing, *etc*.) were more frequent. Generally, concentrations of PCBs were the highest in winter and spring, probably as a result of certain procedures that are more commonly carried out in the cooler seasons (the use of heat exchanger fluids, chemical stabilizers, *etc*.).

### 3.5. Correlations of PCB’s Concentration in Runoff vs. Precipitation

Table 8 shows the average concentrations of PCBs determined in the rainfall and runoff water samples collected from Gdansk Airport area in 2008.

Based on the conducted analysis of rainfall and runoff water samples collected from the area of the airport, the presence of all seven compounds from the group of PCBs (PCB 28, PCB 52, PCB 101, PCB 118, PCB 153, PCB 138, PCB 180), both during the winter and spring season, can be noticed. The concentration levels of all seven investigated compounds in the runoff water samples during the entire research period were significantly higher, when compared with rainfall. In the runoff water samples collected during winter and spring seasons, the concentrations of individual compounds from the group of PCBs were from 1.5 to 13.5 (spring) times greater, when compared with rainfall. The highest concentration levels of the sum of PCBs were determined in the samples collected at the measurement site no. 3 (airport aprons close to the passenger terminal with parked aircraft) in winter, while at the measurement site no. 4 (machinery park) in spring.

The average concentration level of the sum of PCBs calculated for runoff water samples collected from all measurement locations at the airport is comparable to the concentration of the sum of PCBs in the runoff water samples determined during the spring season. However, the average concentration of the sum of PCBs determined in the runoff water samples collected in winter is up to ca. 4.5 times higher in comparison with the concentration of the sum of PAHs determined in the rainfall water samples. It can be concluded that the level of PCB pollution at the airport is highly influenced by both the season, during which the samples are collected, and the sampling point at the airport.

## 4. Summary and Conclusions

Runoff water samples and rainfall from Gdańsk Airport were analysed for their PAH and PCB contents over a range of spatial and temporal scales to determine distributions, trends and possible sources. The results from five airport sites from which 125 samples were collected in 2008–2009 suggest that airport runoff water contains small though significant quantities of PAHs and PCBs. It is of interest to compare the results of this study with published information, which, however, is generally limited to PAH levels in the air and soil at airports. Analysis of PCBs in airport runoff water samples is new. PAH concentrations in airport runoff waters are much higher than those in airport air samples (86–120 ng/m^3^) and are comparable with the values in airport soils (1.7–7,529 ng/kg).

This work is mainly focused on the analysis of runoff water samples, because the composition of such environmental samples contains a sum of pollutants derived both from atmospheric precipitation and washed directly from the airport platform. The runoff water samples are a valuable research material (especially in the case of determination of PAH compounds). The qualitative analysis revealed that 11 of 16 investigated PAH compounds can be detected and determined in runoff waters. In addition, the levels of pollutants from the group of PAHs and PCBs in the runoff water samples were from several to tens times higher compared with the concentrations of the same analytes in the rainfall water samples.

The conducted research studies of both runoff and rainfall water samples collected from the area of the airport indicate a significant impact of the season, during which the samples were collected, on the concentration levels of PAH and PCB pollutants. In winter, the concentration levels of the compounds from the group of PCBs and PAHs determined in both runoff and rainfall water samples are much higher in comparison with the other periods. The significant differences in the concentrations of PAH compounds in winter in the investigated environmental samples result from the fact that winter activity associated with maintaining the security at the airports is much greater than in the other seasons. In the harsh, winter weather conditions, aircrafts and vehicles require a longer period for starting the engines, airplanes also need more power during the takeoff, it is directly associated with burning of more fuel. In the winter season, inspections and repairs of the aircrafts are often carried out, what is connected with the consumption of large amounts of fluids, preservatives and related products, which are often a source of pollution.

Sampling point at the airport also has a significant impact on the level of pollutants present in the rainfall and runoff water samples. The highest concentration levels of pollutants from the group of PCBs and PAHs were determined in the places where there is a heavy traffic associated with the processing of the passengers, in the vicinity of the machinery park, runway and on the airport outskirts, where a large load of pollutants is carried by the movement and maintenance of vehicles leaving and entering the airport.

The sources of PAH emissions were also determined on the basis of the diagnostic ratios of levels of particular PAHs (comparison of calculated ratios with a particular criterion). In 2008 and 2009 pyrogenic sources were dominant in airport runoff water. Analysis of the various ratios showed that incomplete combustion of fuels was the major PAH source for the runoff system in the study area. Understanding the sources and pathways of pollutant transport from airports to environment is fundamental for the protection of ecosystems.

The conclusion to be drawn is that the intensity of air traffic has a significant influence on the amounts of PAHs and PCBs produced at the airport. Generally, PAH and PCB concentrations in airport runoff water in 2008 were much higher than in 2009; this is because in 2009 some 50,000 fewer passengers passed through Gdańsk Airport than in 2008.

The data presented here provide a useful baseline for airports—a source of information essential for assessing the hazard to surface and ground waters in their vicinity. They can also be used in the development of new norms for managing airport infrastructure, the aim of which should be to reduce the impact of airport operations on the biotic and abiotic environment.

## Figures and Tables

**Table 1.** Sampling site description of the Lech Wałesa Airport at Gdańsk Rębiechowo.

**Sample code**	**Comments**
1	Airport apron close to the runway.
2	Service road to the runway for ground staff, fire brigade, rescue teams.
3	Airport aprons close to the passenger terminal with parked aircraft.
4	Machinery park—parking and garages for ground staff, fire brigade and rescue team vehicles.
5	Entry gate to the airport premises.

**Table 2.** Operating conditions of the GC-MS system.

**Condition**	**Runoff water, rain water**
**Compounds**	PAH, PCB
**Injector**	Split/splitless
**Injector volume**	2 μL
**Carrier gas**	Helium—70 kPa
**Detector**	Agilent 5975C inert MSD with electron ionization operating in SIM (selected ion monitoring) mode
**Integration system**	MSD ChemStation
**Column**	Zebron capillary GC column (30 m × 0.25 μm × 0.25 μm; max. temp. 320/340 °C)
**Temperature programme**	40 °C do 120 °C (at 40 °C/min) 120 °C do 280 °C (at 5 °C /min) 280 °C for 17 min and 5 min (PCBs)

**Table 3.** Metrological characteristics of the analytical procedure used during this study.

**Analyte**	**PAH**	**Abbreviation**	**Measuring range (ng/L)**	**LOD (ng/L)**	**LOQ (ng/L)**

			0.02–560	0.025	0.075

1	Naphthalene	NAP	0.034–560	0.034	1,02
2	Acenaphthylene	ACY	0.0041–560	0.0041	0.0123
3	Acenaphthene	ACE	0.0041–560	0.0041	0.0123
4	Fluorene	FLU	0.0018–560	0.0018	0.0054
5	Phenanthrene	PHE	0.0025–560	0.0025	0.0075
6	Anthracene	ANT	0.0078–560	0.0078	0.0234
7	Fluoranthene	FLTH	0.014–560	0.014	0.042
8	Pyrene	PYR	0.028–560	0.028	0.084
9	Chrysene	CHR	0.0022–560	0.0022	0.0066
10	Benzo(b)fluoranthene	BbF	0.014–560	0.014	0.042
11	Benzo(k)fluoranthene	BkF	0.0023–560	0.0023	0.0069
12	Benzo(a)pyrene	BaP	0.0055–560	0.0055	0.0165
13	Benzo(a)anthracene	BaA	0.0017–560	0.0017	0.0051
14	Indeno(1,2,3-cd)pyrene	IP	0.43–560	0.43	1.29
15	Dibenz(a,h)anthracene	DahA	0.014–560	0.014	0.042
16	Benzo(ghi)perylene	B(ghi)PER	0.014–560	0.022	0.066

**Table 4.** Characteristic values of molecular indices for determining the origins of PAHs.

**PAH ratio**	**Sources**		
	**Pyrolytic**	**Petrogenic**	**Other**
NAP/PHE	>1 [43]	<1 [43]	-
PHE/ANT	<10 [14,16,44,45]	>10 [14] >25 [44,45]	4.13 [46] Creosote >15 [16] Crude oil; 50 [47,48] Fuel oil
ANT/(ANT + PHE)	>0.1 [49]	<0.1 [49]	-
BaA/CHR	>1 [50] >0.9 [50]	<1 [50] <0.4 [50]	0.47–0.59 [51,52] Petrol emissions; 1.05–1.17 [51,52] Coal emissions; <1 [50] <0.4 [50] Crude oil
BaA/(BaA + CHR)	>0.2 [53]	<0.2 [53]	
FLTH/PYR	>1 [14]	<1 [14,43]	1.5 [46] Creosote; <1 [14] Crude oil; 0.9 [47] Fuel oil
FLTH/(FLTH + PYR)	0.4–0.5 [53] >0.5 [53]	<0.4 [53]	<0.5 [36,52,54–56] Petrol emissions; >0.5 [52,54–56] Diesel;
IP/(IP + benzo[ghi]perylene)			0.18 [57] Petrol emissions; 0.48–0.57 [43] Coal emissions; 0.56 [57] Coal soot; 0.09 [58] Crude oil

**Table 5.** Percentage relationship between pyrolytic, petrogenic and other sources of PAH emissions at Gdańsk airport in 2008–2009.

**PAH ratio/source**	**Criterion**	**Pyrolytic origin**	
		**2008**	**2009**
NAP/PHE	>1	100%	100%
PHE/ANT	<10	100%	100%
ANT/ANT + PHE	>0.1	100%	100%
BaA/CHr	>1	80%	60%
BaA/BaA + CHr	>0.2	100%	100%
FLTH/PYR	>1	100%	80%
FLTH/PFLTH + PYR	>0.5	0%	0%
		**Petrogenic origin**	
		**2008**	**2009**
NAP/PHE	<1	0%	0%
PHE/ANT	>10	0%	0%
ANT/ANT + PHE	<0.1	0%	0%
BaA/CHR	<1	20%	40%
**PAH ratio/source**	**Criterion**	**Pyrolytic origin**	
		**2008**	**2009**
BaA/BaA + CHr	<0.2	0%	0%
FLTH/PYR	<1	0%	0%
FLTH/PFLTH + PYR	<0.4	20%	20%
		**Other sources**	
		**2008**	**2009**
FLTH/PYR	0.9	0%	20% fuel oil
FLTH/PFLTH + PYR	>0.5	80% diesel	80% diesel
IP/(IP + benzo[ghi]perylene)	0.48–0.57	ND	ND

ND—not detected.

**Table 6.** The average PCB concentration determined in the runoff and rainfall water samples.

**PCB (μg/L)**	**Winter**		**Spring**	

	**Average value of 5 sites from airport**	**Rainfall**	**Average value of 5 sites from airport**	**Rainfall**

PCB28	0.020 ± 0.015	0.004 ± 0.0002	0.055 ± 0.012	0.006 ± 0.0003
PCB52	0.117 ± 0.088	0.056 ± 0.0028	0.136 ± 0.088	0.01 ± 0.0005
PCB101	0.029 ± 0.007	0.018 ± 0.0009	0.050 ± 0.011	0.006 ± 0.0003
PCB118	0.011 ± 0.006	0.005 ± 0.0003	0.016 ± 0.007	0.002 ± 0.0001
PCB153	0.035 ± 0.012	0.014 ± 0.0007	0.017 ± 0.009	0.002 ± 0.0001
PCB138	0.045 ± 0.011	0.017 ± 0.0009	0.030 ± 0.011	0.006 ± 0.0003
PCB180	0.059 ± 0.022	0.043 ± 0.0022	0.025 ± 0.013	0.005 ± 0.0003
∑PCB	0.317 ± 0.098	0.157 ± 0.0079	0.328 ± 0.049	0.036 ± 0.0018

**Table 7.** Average PCB concentrations found in runoff water samples from Gdańsk Airport (Sites 1–5).

**Lp.**	**PCB**	**Concentration (ug/L)**									
		**2008**					**2009**				

		**Site 1**	**Site 2**	**Site 3**	**Site 4**	**Site 5**	**Site 1**	**Site 2**	**Site 3**	**Site 4**	**Site 5**

1	**PCB28**	0.121	0.0279	0.0062	0.0131	0.0192	0.0376	0.0268	0.0040	0.0114	0.0602
2	**PCB52**	0.0955	0.0420	0.186	0.144	0.1819	0.0244	0.0124	0.0008	0.0153	0.0106
3	**PCB101**	0.0403	0.0228	0.0495	0.0562	0.0287	0.0159	0.0126	0.0016	0.0032	0.0325
4	**PCB118**	0.0136	0.0174	0.0150	0.0164	0.0030	0.0458	0.0256	0.0092	0.0088	0.0014
5	**PCB153**	0.0309	0.0058	0.0439	0.0317	0.0193	0.0518	0.0114	0.0096	0.0203	0.0064
6	**PCB138**	0.0410	0.0080	0.0812	0.0397	0.0192	0.0131	0.0147	0.0298	0.0066	0.0088
7	**PCB180**	0.0172	0.0159	0.0577	0.0908	0.0290	0.0424	0.0030	0.0090	0.0099	0.0007

**Total PCB**		0.360	0.140	0.439	0.392	0.300	0.231	0.106	0.064	0.075	0.121

**Table 8.** The average PAH concentration determined in the runoff and rainfall water samples.

**PAH (ng/L)**	**Winter**		**Spring**	

	**Average value of 5 sites from airport**	**Rainfall**	**Average value of 5 sites from airport**	**Rainfall**

**1.naphthalene**	997 ± 89	127 ± 13	971 ± 21	198 ± 11
**2.acenaphthylene**	ND	ND	10 ± 1.3	10 ± 1.0
**3.acenaphthene**	69 ± 25	13 ± 2.6	61 ± 3.2	25 ± 1.2
**4.fluorene**	ND	ND	ND	22 ± 1.3
**5.phenanthrene**	262 ± 44	34 ± 1.7	259 ± 9.1	129 ± 22
**6.anthracene**	124 ± 60	ND	130 ± 11	ND
**7.fluoranthene**	208 ± 89	4 ± 0.88	223 ± 16	153 ± 12
**8.pyrene**	138 ± 54	ND	151 ± 29	118 ± 16
**10.benzo(a)anthracene**	27 ± 11	ND	52 ± 13	73 ± 4.1
**9.chrysene**	41 ± 15	ND	60 ± 8.1	104 ± 10
**11.benzo(b)fluorant-en**	122 ± 54	ND	168 ± 10	221 ± 11
**12.benzo(k)fluorant-en**	ND	ND	22 ± 11	154 ± 8.7
**13.benzo(a)pyrene**	9 ± 1.9	ND	32 ± 15	134 ± 7.9
**14.indeno(1,2,3,-cd)pyrene**	3 ± 1.0	ND	11 ± 3.3	137 ± 7.8
**15.dibenzo(a,h)antracene**	ND	ND	ND	ND
**16.benzo(g,h,i)perylene**	ND	ND	21 ± 15	127 ± 6.9
**∑PAHs**	2000 ± 459	179 ± 39	2172 ± 111	1604 ± 80
